# Supplementary material for: Active site specificity profiling datasets of matrix metalloproteinases (MMPs) 1, 2, 3, 7, 8, 9, 12, 13 and 14
Source: Data Brief. 2016 Feb 22;7:299–310. doi: 10.1016/j.dib.2016.02.036 (PMC4777984; doi:10.1016/j.dib.2016.02.036)
Supplement: Supplementary file 10 — Supplementary material [file mmc10.zip › WebPICS_hMMP1_T_1%/P3.html]

 

PICS results


|  |  |
| --- | --- |
| **P3\_A**  32 in 277 sites   11.6 %    effects > 10 perc. pnts.  (vice-versa in brackets)  P3prime\_E: 10.1 (13.4) |  |
| **P3\_C**  12 in 277 sites   4.3 %    effects > 10 perc. pnts.  (vice-versa in brackets)  P2\_L: 36.6 (11.9)   P3prime\_S: 33.4 (17.4) |  |
